# The Treatment of Puerperal Sepsis in General Practice

**Published:** 1914-04-18

**Authors:** A. Knyvett Gordon

**Affiliations:** sometime Medical Superintendent of Monsall Hospital


### THE TREATMENT OF PUERPERAL SEPSIS IN GENERAL PRACTICE.

By A. KNYVETT GORDON, M.B., B.C.Cantab., sometime Medical Superintendent of Monsall Hospital.

Quite recently, in the course of some conversation with a brother practitioner, it was pointed out to me that it was very generally believed that the methods advocated in text-books and original papers for the treatment of puerperal sepsis were but rarely possible or advisable in general practice. I thought there was some excuse for this view, though I did not agree with it, and I therefore wrote the following notes on a few of the points which seemed to me to be of importance in dealing with a disease which is a source of so much anxiety when it occurs—as it occasionally does—in the clientèle of a practitioner who conducts his obstetric operations with due attention to asepsis. The man who does not is that fortunate person who "never has a case of puerperal fever"—or calls it influenza.

Though I do not wish here to discuss the difficult questions connected with the causation of the disease, a few words on its pathology are necessary in order to clear the ground. We are all agreed that "Puerperal Fever is Wound Fever," and that while the uterus is sterile during pregnancy and at the time of delivery, the vagina is teeming with organisms, especially *Bacillus coli* and streptococci of various kinds. When these come in contact with the raw surface at the placental site puerperal sepsis may result. The infecting organisms are certainly not always introduced from without; a good example of this is seen in the cases where sepsis follows the introduction of a carefully disinfected and rubber-gloved hand into the uterus for the removal of an adherent placenta, it being almost impossible in an emergency to sterilise the vagina through which the hand has to pass.

I would here add that, in my view, puerperal sepsis does not often arise from infection of a perineal laceration only. In the cases of general sepsis, where this latter has been present, I have never failed to find, on bacteriological examination, contamination of the uterine cavity also. The explanation of this lies in the fact that many of the vaginal organisms are in reality protective, and that the tissues of the perineum have from constant association with bacteria of all kinds acquired a sort of immunity.

The practical point here is that when we have to deal with a case of systematic infection, we must concentrate our attention on the cavity of the uterus. From this organ the bacteria can reach the system by one, or all, of three ways. They may travel straight up the Fallopian tubes, giving rise either to pyosalpinx, if some protective sealing of the abdominal ends occurs, or to general peritonitis, if the resistance of the patient is unable to effect this, or we may have infection of the blood in the uterine sinuses with local thrombosis (in the periuterine and ovarian veins), or rapidly fatal general septicæmia, if the virulence is too great or the resistance too poor for thrombosis to occur. Lastly, we get the milder cases where the pelvic connective tissue and peritoneum become involved in inflammation, which usually goes on to suppuration, with or without adhesions, that makes the patient a chronic pelvic invalid, and, in the poorer classes, results in an addition to the pathetic crowd that attends the gynæcological out-patient department of our hospitals.

We come now to the practical side of the problem—namely, what to do with a patient who has a rise of temperature after delivery which we suspect to be due to puerperal sepsis. In the first place, we must not wait—or rely on general measures alone—until we are certain of our diagnosis before attacking the uterus. That way tragedy lies. The guide for action should be the state of the pulse, which is practically always quickened *ab initio* in all but the very mild cases of infection. No harm whatever results if we disinfect the uterus in cases where the pyrexia is really due to absorption from the alimentary canal, and if we omit to seize the precious moments at the beginning of a serious infection from the uterus, by delaying until the presence of rigors or a distended abdomen establish the diagnosis, we may find ourselves subsequently signing the death ce[illegible]ficate of a preventible disease. *It is, in the great majority of cases, impossible to be sure of the diagnosis of puerperal infection until the mischief has spread beyond the uterus.*

We disinfect the uterus, then, but we must do it adequately, and here a protest must be entered against the common error of thinking that the use of the intrauterine douche is all that is necessary. In reality it is only useful in cases of pure sapræmia, where the pyrexia is due to absorption of toxins from *loose* decomposing uterine *contents.* It can have little or no action on the uterine "wound," for if a section of the placental site in a fatal case be examined, the organisms are found not only on the surface but below it, and it is difficult to imagine that merely playing on the surface with any antiseptic solution of a strength which can be used with safety can do the active organisms in the uterine tissues much harm. Moreover, the procedure is not without danger on account of the risk of washing infective matter up the Fallopian tubes. I have, in fact, seen three cases in which the patient was admitted to hospital with general peritonitis, which followed on the use of the intrauterine douche for symptoms which were previously not severe.

It is far better to begin by exploring the uterus. For this purpose a general anæsthetic is not necessary; if a moderate dose of hot brandy or whisky and water be given one hour before the exploration, followed in half an hour by a hypodermic injection of a quarter of a grain of morphia, or by one-sixth of a grain of morphia with one-hundredth of a grain of hyoscin, the patient is quite analgesic, and in practice does not usually remember the details of the procedure subsequently.

The patient is then placed in the lithotomy position, and the cervix exposed with a speculum and drawn down and steadied with vulsella. Dilatation is not usually necessary, but can be effected quite easily with Hegar's instruments if there is not sufficient room for the examining finger, which is then introduced. For the adequate exploration of every part of the uterine wall it is necessary that the fundus should be pushed down by a hand placed on the abdominal wall. Sometimes loose or partially detached masses of placental tissue can be felt, and it then usually suffices to swab the whole lining of the uterine cavity after they have been gently detached and removed. For this purpose gauze soaked in some reliable non-toxic antiseptic (I have had the best results with undiluted Izal, but formalin and glycerine or pure peroxide of hydrogen have each their advocates) is wrapped round a pair of forceps so as to form a large soft mop, and with this the endometrium is methodically rubbed; about a dozen swabs are usually necessary for the purpose. No douche is given, nor should the uterine cavity be packed, but a large plug of gauze soaked in glycerine is left in the vagina to be removed in twenty-four hours. When placental masses are found the case usually does well.

But it oftens happens that one of two other conditions is found on exploration. Either the uterine wall is smooth, and there is apparently nothing the matter locally except that the uterus is larger than it should be, and dilated and toneless, or it may be that the whole uterine wall is soft and boggy, so that holes can be made in it with the examining finger, unless the utmost gentleness is used. We will take the latter class of case first. Here simple swabbing does not in practice usually suffice, and curetting is required. In my opinion this should be done with a light sharp curette, and not with the much more dangerous blunt instrument, for which some force is required. What is wanted is to remove the septic endometrium down to the muscle, and then to swab the raw surface as before, using soft mops, much antiseptic, and very little force. The flushing curette has been recommended, but it is a cumbersome instrument, and delicacy of touch is almost impossible. Without careful swabbing of the raw surface, any form of curetting is quite unjustifiable. In these cases, the uterus usually begins to contract when the swabbing is commenced; if it does not, the outlook is bad. In the other type of case, where the endometrium is smooth, the value of curetting is not so marked. Generally these cases have streptococci in the circulating blood when the temperature first rises, and death from general septicæmia must be expected. But my own experience leads me to believe that it is better to curette and swab these cases than to rely on general measures only. The problem is, however, a difficult one, and each case must be judged on its merits. *Post-mortem* one always finds the uterine wall full of micro-organisms when stained sections are examined, though the endometrium has previously *felt* normal, so local treatment would appear to have some theoretical justification also.

Coming now to general measures, there are some which are undoubtedly valuable and others which are, I believe, almost completely useless, though they are copied from text-book to text-book with unfailing regularity. In the former class I would place, firstly, the introduction by one means or another of large quantities of fluid. If the patient can drink, it suffices to administer at least five pints of water or thin barley water in the twenty-four hours. Such cases usually recover. Other methods are subcutaneous infusion of saline solution, the fluid being run in gradually underneath the breasts with a serum needle connected to a syphon douche, and rectal irrigation with the same solution. I believe the subcutaneous method to be the better both in theory and in practice, and it is much the more easy to perform. Sometimes large doses of antistreptococcic serum added to the subcutaneous saline injections are valuable, but I have found the sera without the saline quite useless.

Next in value I would place calomel, preferably given in grain doses every hour for four or five hours, and repeated after a few hours intermission. Fortunately, pain is not often a prominent feature in puerperal sepsis; the administration of opium is, of course, to be avoided whenever possible, as it makes the early diagnosis of peritonitis—should this unfortunately supervene—impossible.

In almost all the text-books the administration of quinine is advised, and in many reduction of the temperature by means of the coal-tar antipyretics and salicylates is also suggested. In my hands quinine has been utterly useless, though I have seen it given very frequently; in fact, it used to be quite common to find patients certified to be suffering from puerperal fever admitted to hospital with intense tinnitus from its previous employment. I have never satisfied myself that this drug has any beneficial action in septic absorption from the pelvic organs. Chemical antipyretics of the coal-tar series are often dangerous, and there is really not much to be gained by treating the patient's temperature chart! If the pyrexia *per se* is having a bad effect on the patient, tepid or cold sponging is much more suitable.

It will be seen that all the methods I have advocated are such as can be adopted quite easily in general practice; for the exploration and swabbing of the uterus, even if curetting is also resorted to, a general anæsthetic is not necessary, and all the required manipulations can be conducted on the patient's own bed without skilled assistance. Even if it be found necessary to use saline injections subcutaneously, the simple apparatus (once the flow is started) can be managed quite easily by an intelligent relative of the patient.

I have purposely not discussed such measures (as the treatment of peritonitis) which require the help of a surgeon skilled in abdominal technique.

